# Orbital Fractures at a Level 1 Trauma Center: A Retrospective Epidemiological Analysis of 1489 Consecutive Fracture Records

**DOI:** 10.3390/jcm15207764

**Published:** 2026-10-09

**Authors:** Tal Capucha, Ahmad Hija, Chaim Ohayon, Fadi Abu Shqara, Andrei Krasovsky, Amir Bilder, Amir Wolff, Omri Emodi

**Affiliations:** 1 Department of Oral and Maxillofacial Surgery, Rambam Health Care Campus, Haifa 3109601, Israel; 2 Ruth & Bruce Rappaport Faculty of Medicine, Technion-Israel Institute of Technology, Haifa 3200003, Israel

**Keywords:** orbital fracture, blow-out fracture, orbital floor, epidemiology, inferior rectus muscle, maxillofacial trauma, defect morphometry, level 1 trauma center, content herniation, diplopia

## Abstract

**Objectives:** Comprehensive epidemiological data on orbital fractures from large full-spectrum series remain scarce. We describe the demographics, mechanisms, fracture anatomy, CT-derived defect morphometry, and clinical findings at presentation of orbital fractures managed at a level 1 trauma center. **Methods:** We retrospectively analyzed 1489 consecutive CT-confirmed orbital fracture records managed at Rambam Health Care Campus, Haifa, Israel, between September 2001 and November 2023. Demographics, mechanism, fracture location, laterality, CT-derived defect dimensions, orbital content herniation, inferior rectus muscle (IRM) involvement on CT, diplopia, concomitant facial fractures, and timing of repair were analyzed. The analysis was planned as descriptive; a single pre-specified comparison (age between sexes) was tested, and no inferential modelling of treatment decisions or outcomes was undertaken. **Results:** The cohort was 78.6% male (M:F 3.7:1), with a mean age of 36.5 ± 20.0 years; patients aged 18–29 years predominated (33.5%). Females were older than males (46.3 ± 24.6 vs. 33.9 ± 17.7 years; *p* < 0.001). Falls (33.0%) and assault (26.1%) were the principal mechanisms. The orbital floor was involved in 87.7% of fractures (isolated in 58.6%); mean sagittal and coronal defect diameters were 13.7 ± 6.1 and 13.3 ± 5.1 mm. Content herniation was present in 55.1%, IRM involvement on CT in 14.7%, and diplopia in 17.6%; concomitant facial fractures occurred in 27.9%, and 72.9% underwent repair, at a median of 2 days. **Conclusions:** In one of the largest single-center orbital fracture cohorts reported, a young-adult male, floor-dominant profile predominated. Herniation, radiological muscle involvement, diplopia, and associated facial fractures were common, indicating the functional and diagnostic complexity of this injury pattern. These descriptive findings characterize the population presenting to a level 1 center; they do not evaluate management strategies and should not be read as establishing surgical indications, thresholds, or optimal timing.

## 1. Introduction

Orbital fractures are a common and clinically consequential component of maxillofacial trauma. The orbit, a pyramidal cavity bounded by seven bones, houses the globe, optic nerve, and six extraocular muscles within periorbital fat and fascia. Its anatomical complexity makes fractures consequential beyond the structural breach: disruption of the orbital walls may produce enophthalmos, hypoglobus, diplopia from muscle entrapment or edema, infraorbital nerve hypesthesia, and, in severe presentations, reduced visual acuity or optic neuropathy [1,2,3]. The principal vulnerabilities lie at the orbital floor, formed by the thin infraorbital plate of the maxilla, and at the medial wall (lamina papyracea of the ethmoid), both prone to fracture through hydraulic or buckling force transmission during blunt periorbital impact [4,5].

Three variables carry particular weight in the assessment of these injuries, and each motivated a specific objective of this study. Defect morphometry on CT is the principal objective anatomical parameter available at presentation, and linear and area-based measurements have been used to structure operative decision-making and reconstruction planning [6,7,8,9]; how these dimensions are distributed across an unselected presenting population is not well described. Inferior rectus muscle (IRM) involvement matters because sustained incarceration may cause ischemia and fibrosis with lasting motility restriction, yet published prevalences derive almost entirely from operative series and therefore describe a selected population [10,11]. Timing of repair is the variable most often invoked in management discussion, but its distribution in routine practice across an entire institutional cohort is rarely reported.

Published epidemiological data remain heterogeneous. Most institutional series report fewer than 400 patients [12,13], limiting characterization of clinically important subgroups. National database analyses [3,14] provide large denominators but lack imaging-based granularity. The European Maxillofacial Trauma (EURMAT) project [14], spanning 13 countries, described broad facial fracture patterns without orbit-specific anatomical or functional detail. The largest published institutional cohort of orbital floor fractures, 1594 surgically treated patients from Frankfurt [2], excluded non-operated cases and therefore overrepresents severe defects. A recent systematic review of CT parameters in pure orbital wall fractures [9] found the evidence linking specific measurements to treatment choice and outcome to be inconsistent, and identified the absence of large full-spectrum cohorts with standardized morphometry as a principal gap.

Rambam Health Care Campus is the level 1 trauma and maxillofacial referral center for northern Israel, a demographically diverse region of approximately two million inhabitants with a substantial burden of road traffic injury, interpersonal violence, and ballistic trauma. A systematically maintained institutional database allows retrieval of orbit-specific variables, including CT-derived defect dimensions, IRM status, and timing of repair, that are rarely reported at this scale.

This study reports the epidemiological, anatomical, and clinical characteristics of 1489 consecutive orbital fracture records. Specific objectives were to: (1) characterize the demographic profile; (2) describe mechanism of injury, fracture location, laterality, and defect morphometry; (3) document the prevalence of IRM involvement on CT, content herniation, and diplopia at presentation; and (4) describe the distribution of timing of repair in the operative subgroup. The study was designed as a descriptive epidemiological analysis. It was not designed to compare management strategies or to evaluate outcomes, and the data reported here establish a denominator for the outcome-focused analyses planned from this cohort.

## 2. Materials and Methods

### 2.1. Study Design and Setting

A retrospective cohort study was conducted at the Department of Oral and Maxillofacial Surgery, Rambam Health Care Campus, Haifa, Israel. The study was designed and reported in accordance with the Strengthening the Reporting of Observational Studies in Epidemiology (STROBE) guidelines [15]. Ethical approval was obtained from the Rambam Institutional Review Board (Protocol No. 0191-26-RMB-D; approved 23 June 2026), and the study was conducted in accordance with the Declaration of Helsinki. Informed consent was waived given the retrospective, anonymized design.

### 2.2. Patient Selection and Referral Pathway

**Figure 1:**
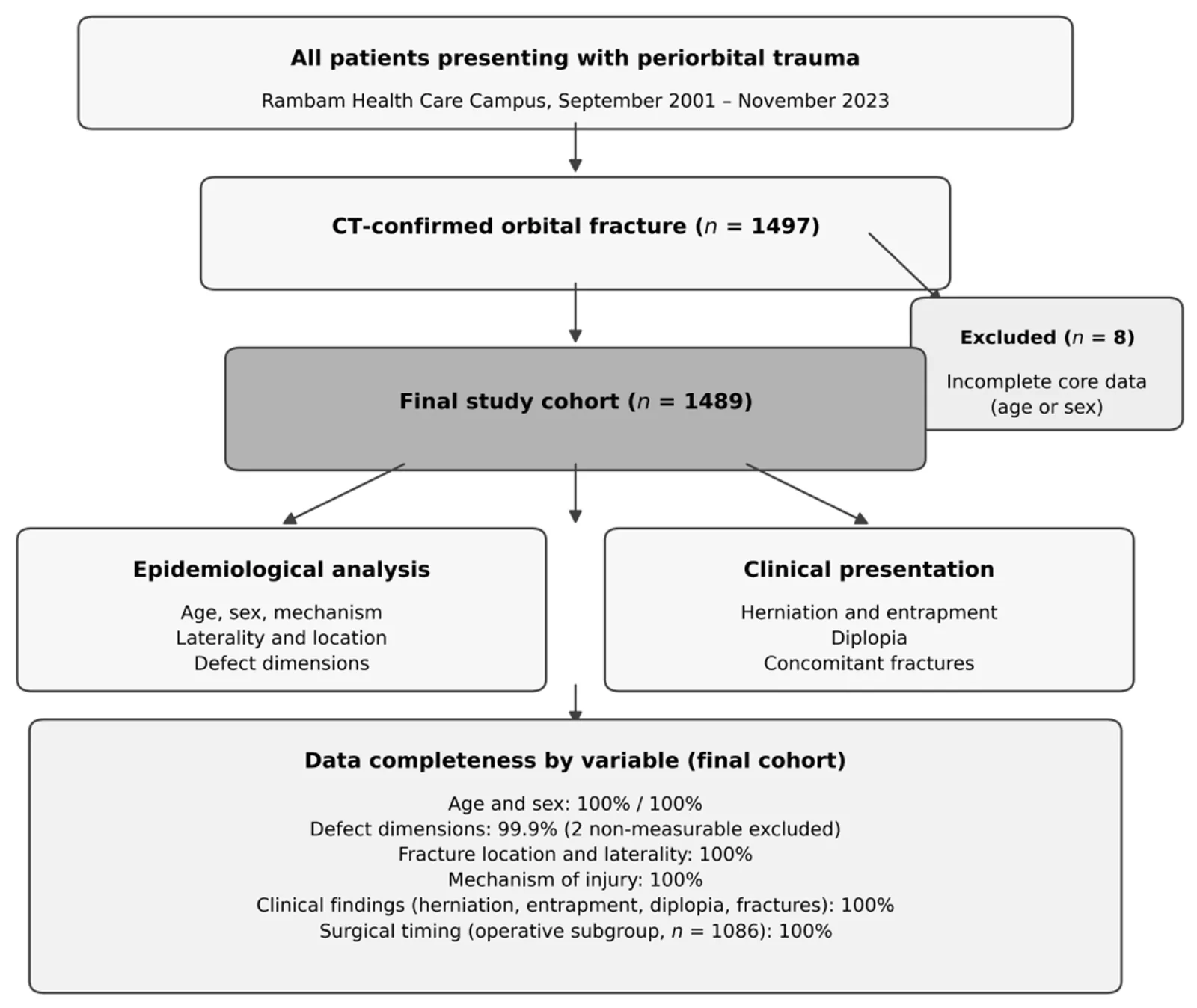
STROBE-compliant patient inclusion flowchart, with data completeness summarized by variable category. IRM, inferior rectus muscle.

All patients presenting between 8 September 2001 and 28 November 2023 with an orbital fracture confirmed by multiplanar computed tomography (CT) of the facial skeleton (coronal, axial, and sagittal planes; bone and soft-tissue windows) were eligible. Patients were excluded if CT confirmation was unavailable or if core demographic data (age or sex) were absent. No restriction was applied with respect to age, fracture severity, laterality, mechanism, or management strategy. A STROBE-compliant inclusion flowchart with a data completeness summary is presented in Figure 1.

The referral pathway is relevant to the interpretation of the operative proportion reported below. The department is the regional maxillofacial referral service, and the database captures patients referred to or managed by that service rather than every patient with a radiologically detected orbital fracture in the catchment area. Patients with incidental, minimally displaced fractures identified on head CT performed for other indications, and managed entirely within emergency medicine or ophthalmology without maxillofacial referral, are not consistently represented. The cohort therefore spans both operative and non-operative management but is drawn from a referred population, and the 72.9% operative proportion should be read in that light rather than as the operative rate among all orbital fractures occurring in the region.

The unit of analysis is the database record rather than the unique patient. A patient may contribute more than one record where separate clinically distinct management or procedural episodes were documented, so the number of unique individuals represented is smaller than 1489. All counts and proportions reported below are record-level. Because records from the same patient are not statistically independent, no inferential modelling was performed beyond the single pre-specified comparison described in Section 2.5, and any subsequent analysis of these data will require clustering by patient identifier.

### 2.3. Data Collection and Variable Definitions

Variables extracted from the institutional electronic clinical database included: age and sex; mechanism of injury (fall, assault/interpersonal violence, motor vehicle accident [MVA], sports/recreational injury, workplace accident, ballistic injury, other blunt trauma); fracture laterality; fracture location (orbital floor [isolated], floor plus inferior orbital rim, floor plus medial wall, orbital roof, medial wall [isolated], multi-wall/other); maximum sagittal and coronal defect diameters; orbital content herniation on CT; IRM involvement on CT; diplopia at clinical assessment; concomitant facial fractures; and time from diagnosis to surgical repair in days for the operative subgroup. Sagittal defect diameters were categorized as small (<10 mm), medium (10–20 mm), or large (>20 mm), consistent with prior series [2,12].

IRM involvement was defined radiologically, as CT evidence of muscle displacement into or incarceration within the fracture line or maxillary sinus. This is a radiological descriptor and is not synonymous with clinically or surgically confirmed entrapment. Forced-duction testing was not performed systematically and was not recorded in the database, and dynamic motility assessment was documented inconsistently and could not be correlated with the CT finding. The same radiological definition was applied to adult and pediatric records; no separate pediatric criterion was used. Throughout this manuscript the finding is therefore reported as IRM involvement on CT rather than as entrapment, and its prevalence should be interpreted accordingly.

### 2.4. CT Morphometry

Maximum sagittal and coronal defect diameters were measured on standardized multiplanar reconstructions in the bone window. The sagittal diameter was taken as the maximum anteroposterior extent of the bony defect on the sagittal reconstruction through the center of the defect; the coronal diameter was taken as the maximum mediolateral extent on the coronal reconstruction at the level of the greatest defect width. Measurements were made with the electronic calliper of the institutional picture archiving and communication system.

Measurements were performed by two maxillofacial surgeons with dedicated experience in orbital trauma, working from the archived studies, and were recorded during database curation rather than at the time of clinical care. Scanner hardware and acquisition protocols changed over the 22-year study period; slice thickness was not uniform across the whole period and was not retrievable for every study. Formal intraobserver and interobserver reliability testing was not performed, and no reliability coefficient can be reported for these measurements. This is a substantive limitation of the morphometric data and is addressed in Section 4.6.

Estimated defect area was modelled as an ellipse, (π/4) × sagittal × coronal diameter. Orbital wall defects are irregular three-dimensional surfaces, and no two-diameter formula reproduces their true area; the elliptical model was chosen because it is reproducible from two routinely recorded linear measurements and is less biased upward than the rectangular product of the same diameters. It should be read as a standardized size index for comparison within this cohort, not as a measurement of true defect surface area, and areas derived by different formulae are not directly comparable across studies.

### 2.5. Statistical Analysis

All analyses were performed using IBM SPSS Statistics, version 32.0 (IBM Corp., Armonk, NY, USA). Continuous variables are reported as mean ± standard deviation (SD), median, and range, with the interquartile range (IQR) where distributions were right-skewed. Categorical variables are reported as absolute frequencies and proportions. The analysis was planned and executed as descriptive. A single pre-specified comparison, age between sexes, was assessed using the Mann-Whitney U test, with two-sided *p* < 0.05 considered significant. No further hypothesis testing was performed: associations between sex, age, and mechanism, and between fracture characteristics and management, were not formally analyzed, and statements about such relationships are accordingly presented as description or as reference to previous literature rather than as findings of this study. Missing data are reported per variable; no imputation was performed.

## 3. Results

### 3.1. Patient Demographics

**Figure 2:**
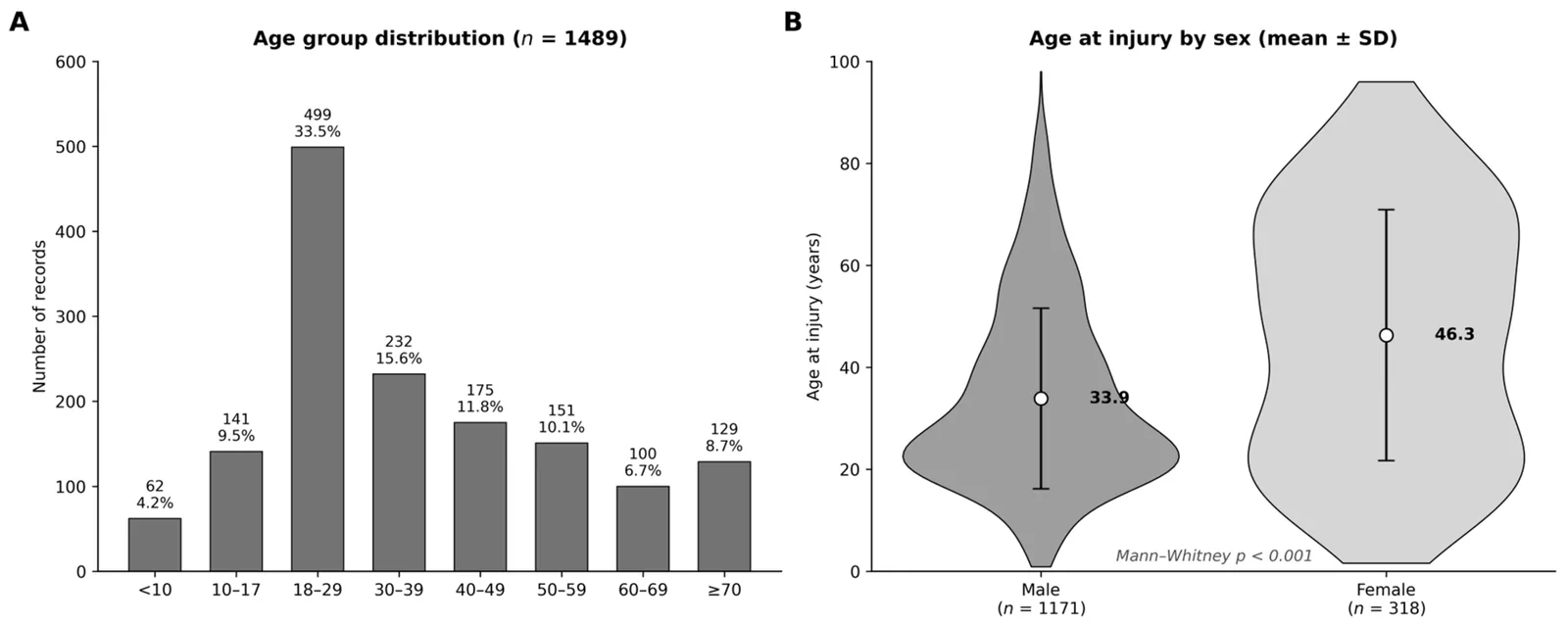
Age and sex distribution of the cohort. (**A**) Age group distribution (*n* = 1489); values above bars are absolute counts and percentages. (**B**) Violin plot comparing age at injury between male and female patients; circles indicate group means, error bars ±1 SD.

A total of 1489 records were included; age and sex were documented for all. The demographic profile is presented in Table 1 and Figure 2.

Mean age was 36.5 ± 20.0 years (median 31; IQR 21–51; range 1–98). Males constituted 78.6% (*n* = 1171), giving an M:F ratio of 3.7:1. The largest single age group was 18–29 years (33.5%; *n* = 499), with a smaller elevation among patients aged ≥70 years (8.7%; *n* = 129); formal testing for bimodality was not performed, and the distribution is therefore described rather than classified. Pediatric patients (<18 years) comprised 13.6% (*n* = 203) and patients aged ≥65 years 11.3% (*n* = 168).

Male patients were younger than female patients at injury (33.9 ± 17.7 vs. 46.3 ± 24.6 years; difference 12.4 years; Mann-Whitney U test, *p* < 0.001). This was the only pre-specified comparison in the study; the distribution of mechanisms by sex was not tested.

### 3.2. Mechanism of Injury

**Figure 3:**
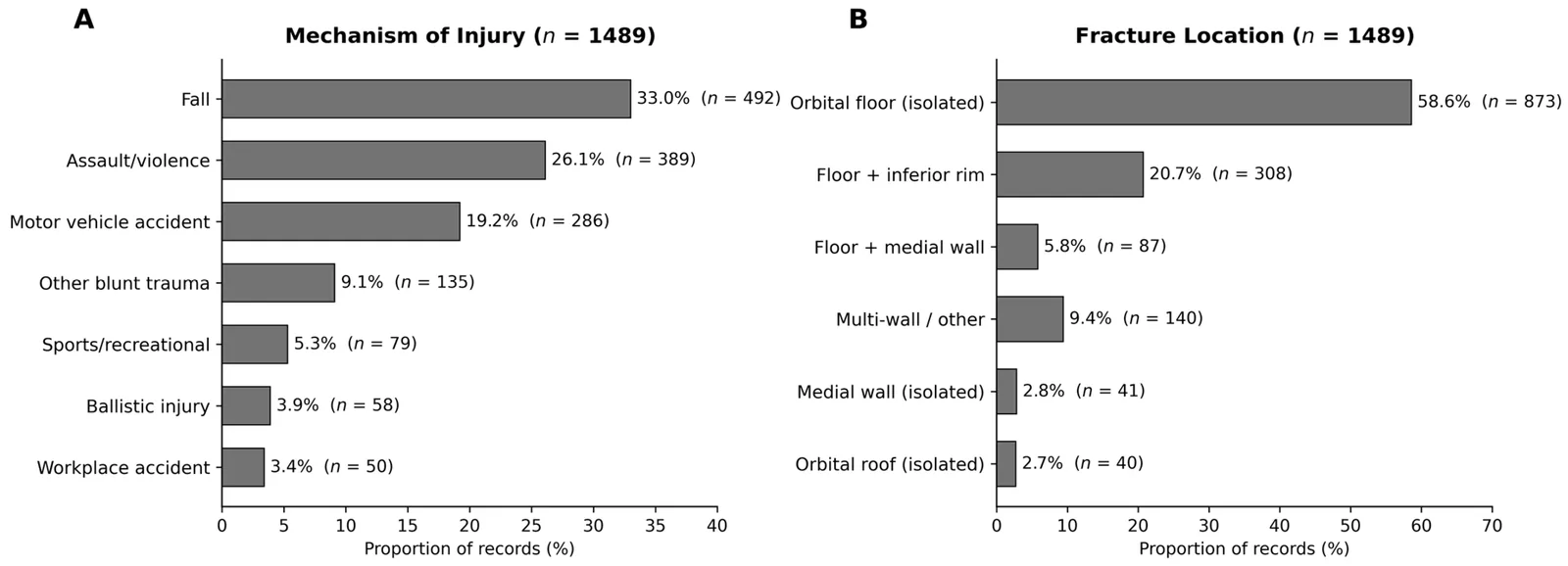
(**A**) Mechanism of injury (*n* = 1489). (**B**) Distribution of fracture locations (*n* = 1489). MVA, motor vehicle accident.

Mechanism was recorded for all 1489 records. Falls were most frequent (33.0%; *n* = 492), followed by assault or interpersonal violence (26.1%; *n* = 389), MVA (19.2%; *n* = 286), other blunt trauma (9.1%; *n* = 135), sports and recreational injury (5.3%; *n* = 79), ballistic injury (3.9%; *n* = 58), and workplace accidents (3.4%; *n* = 50). The distribution is shown in Figure 3A. Mechanism was not cross-tabulated against sex or age group, and no association between mechanism and demographic characteristics was tested.

### 3.3. Fracture Location, Laterality, and Defect Morphometry

Laterality was recorded for all records; left-sided fractures predominated (54.6%; *n* = 813) over right-sided (43.7%; *n* = 651), with bilateral injuries in 1.7% (*n* = 25). The orbital floor was involved in 87.7% (*n* = 1306). Isolated floor fractures were the most frequent pattern (58.6%; *n* = 873) (Figure 3B), followed by floor with inferior rim (20.7%; *n* = 308) and floor with medial wall (5.8%; *n* = 87). Isolated medial wall (2.8%; *n* = 41) and isolated roof (2.7%; *n* = 40) fractures were less common, and 140 records (9.4%) involved multi-wall or other patterns, including 16 in which the location was not specified.

**Figure 4:**
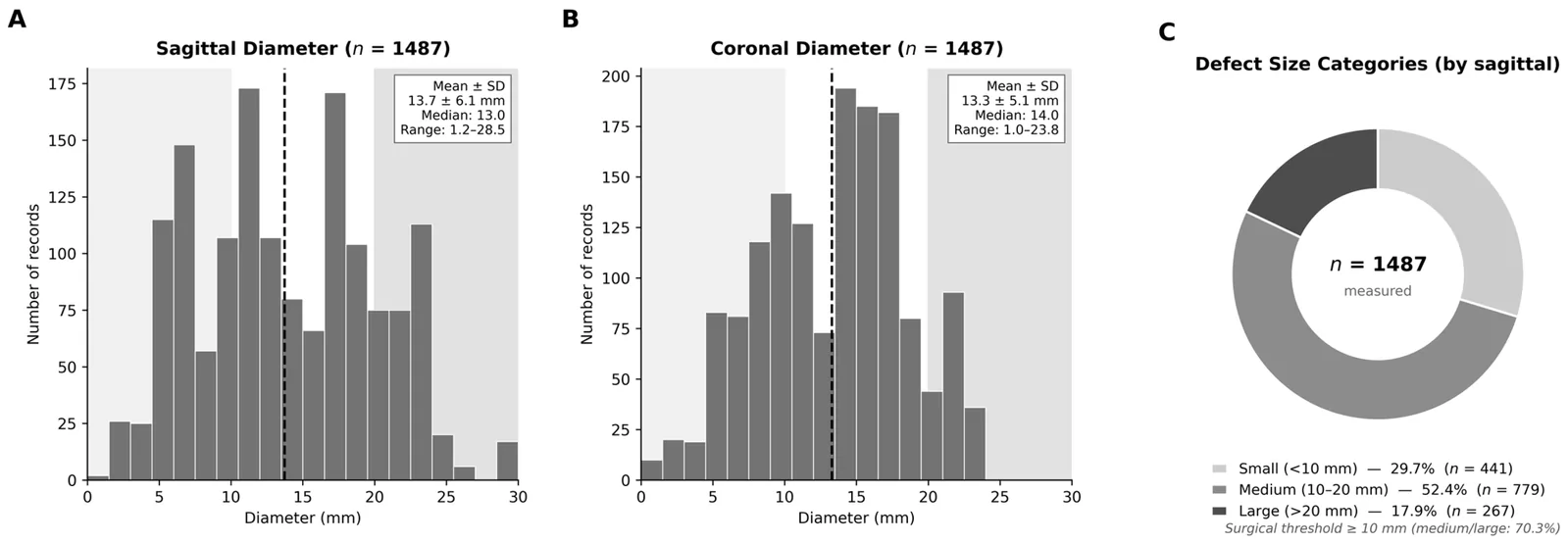
Distribution of orbital defect dimensions on CT. (**A**) Sagittal diameters (*n* = 1487); dashed line indicates the mean, shaded zones the size categories. (**B**) Coronal diameters (*n* = 1487). (**C**) Proportional breakdown of size categories by sagittal diameter.

Sagittal and coronal defect diameters were measured in 1487 records (99.9%; two non-measurable fractures excluded). Mean sagittal diameter was 13.7 ± 6.1 mm (range 1.2- 28.5) and mean coronal diameter 13.3 ± 5.1 mm (range 1.0–23.8). Estimated elliptical defect area was 149.9 ± 94.0 mm^2^. By sagittal diameter, defects were small (<10 mm) in 29.7%, medium (10–20 mm) in 52.4%, and large (>20 mm) in 18.0% (Figure 4). Defect dimensions were not compared between operatively and non-operatively managed patients, and no threshold analysis was undertaken.

### 3.4. Clinical Findings at Presentation

**Figure 5:**
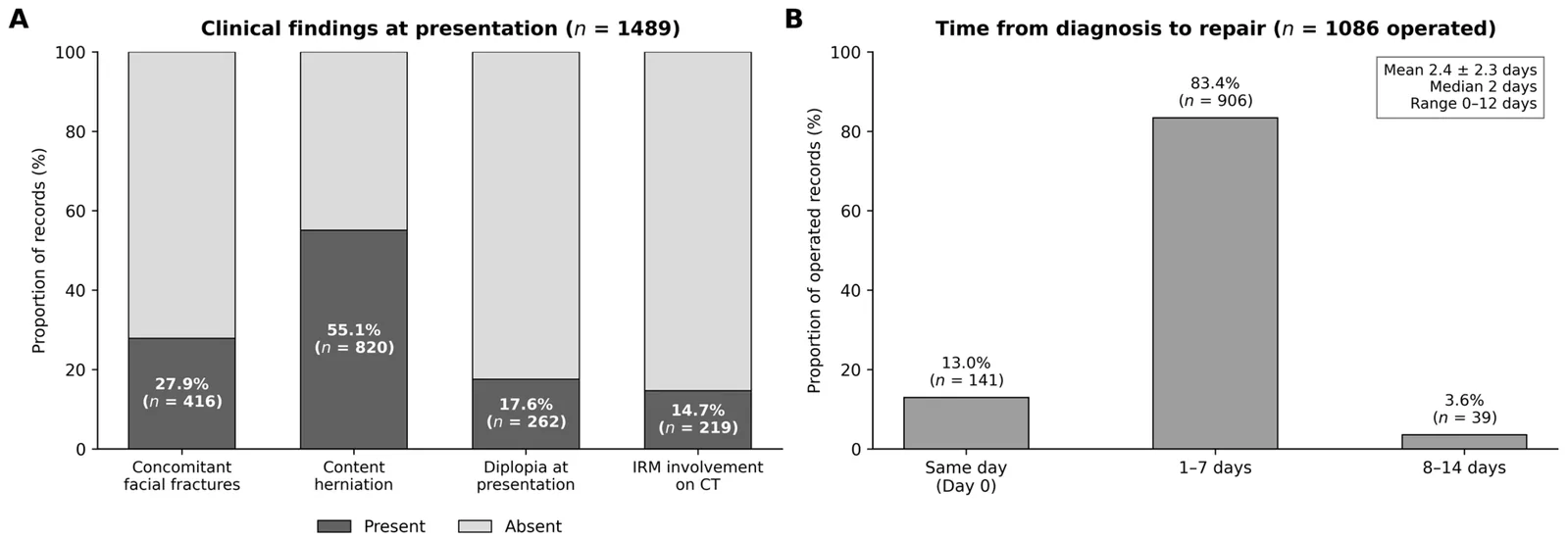
(**A**) Prevalence of clinical findings at presentation among all 1489 records; dark bars indicate presence, light bars absence. (**B**) Distribution of time from diagnosis to repair among the 1086 operated records. IRM involvement on CT is a radiological descriptor (Section 2.3). CT, computed tomography; IRM, inferior rectus muscle.

Orbital content herniation was identified on CT in 820 records (55.1%). IRM involvement on CT, as defined in Section 2.3, was present in 219 records (14.7%). Binocular diplopia at presentation was documented in 262 records (17.6%). Concomitant facial fractures were documented in 416 records (27.9%), most often zygomatic complex and nasal fractures. These data are shown in Figure 5A and summarized in Table 2. Diplopia and radiological IRM involvement were not cross-tabulated, and the concordance between them was not assessed.

### 3.5. Management and Timing of Repair

Of the full cohort, 1086 records (72.9%) underwent surgical repair and 403 (27.1%) were managed non-operatively. The database records the management received but not the reasoning behind each decision; indications were not recorded in a structured field and could not be reconstructed retrospectively. No comparison of clinical or morphometric characteristics between the operative and non-operative groups was performed, and the factors associated with the decision to operate cannot be determined from these data.

Among operated patients, the mean interval from diagnosis to repair was 2.4 ± 2.3 days (median 2; range 0–12). Repair was performed on the day of diagnosis in 141 records (13.0%), within 1–7 days in 906 (83.4%), and at 8–14 days in 39 (3.6%); no repair beyond 12 days was recorded. The reasons for differences in timing, which may include clinical urgency, theatre availability, patient transfer, and management of concurrent injuries, were not recorded and cannot be inferred. Postoperative outcomes were not evaluated in this study, and these timing data therefore describe departmental practice without establishing any relationship between timing and result. The distribution is shown in Figure 5B.

## 4. Discussion

This retrospective cohort of 1489 consecutive orbital fracture records is among the largest single-center institutional series in the contemporary literature, exceeded in scale only by national database analyses that lack imaging-based granularity [3,14]. The Frankfurt series [2] reported 1594 patients but was confined to surgically treated cases and therefore overrepresents severe defects. The present cohort includes both operative and non-operative patients and so describes a broader clinical spectrum, although, as set out in Section 2.2, it remains a referred population. The following discussion is confined to what a descriptive design can support; where explanatory mechanisms are invoked, they derive from the existing literature and were not tested here.

### 4.1. Demographic Profile

The demographic profile, 78.6% male with an M:F ratio of 3.7:1 and the largest age group at 18–29 years, is consistent with international series from European and North American settings [1,2,3,14]. Gassner et al. [1] reported an M:F ratio of 2.1:1 across all craniomaxillofacial fractures in the Innsbruck 10-year series; orbit-specific analyses consistently yield higher ratios. The 3.7:1 ratio observed here is toward the upper end of reported values, which may reflect the trauma load of a level 1 center serving a catchment in which assault (26.1%) and ballistic injury (3.9%) together account for 30% of mechanisms, although this interpretation was not tested.

Male patients were 12.4 years younger than female patients at injury. In the wider literature this pattern is attributed to a predominance of assault, sport, and road traffic mechanisms among younger men and of falls among older women [3,14]. The present study did not analyze mechanism by sex or by age, and this explanation is therefore offered as context from previous work rather than as a finding of this cohort. Comorbidity, bone quality, frailty, and anesthetic risk were not recorded and cannot be commented on. What can be stated is that 11.3% of this cohort were aged ≥65 years, so a substantial minority of patients presenting with orbital fractures at this center are elderly.

### 4.2. Mechanisms and Fracture Anatomy

Falls were the most frequent mechanism (33.0%), displacing assault from the leading position reported in the operative-only Frankfurt series [2] and corresponding to the EURMAT multicentre pattern [14]. A plausible contributor is the inclusion of non-operated and older patients in the present cohort, though this was not formally examined. Ballistic injuries accounted for 3.9%, exceeding rates reported in most Western European institutional series. Their anatomical and management complexity was not characterized in this study: soft-tissue injury, neurosurgical involvement, and ophthalmological co-management were not recorded variables, and no claim about the severity profile of this subgroup is made here.

The predominance of the orbital floor (58.6% isolated, 87.7% when all floor-involving patterns are included) is consistent with the biomechanical properties of the infraorbital plate. Both the hydraulic mechanism, in which sudden intraorbital pressure increase compresses the floor until it yields [4], and the buckling mechanism, in which force transmitted along the orbital rim causes failure at the most compliant point [5], target this site. Ahmad et al. [4] showed in cadaveric experiments that hydraulic forces generate larger, more posterior defects involving floor and medial wall, a pattern compatible with the 5.8% floor-plus-medial-wall frequency observed here. Orbital roof fractures accounted for 2.7%.

### 4.3. Defect Morphometry

Mean defect dimensions (sagittal 13.7 mm, coronal 13.3 mm, estimated elliptical area 149.9 mm^2^) are consistent with a cohort that includes non-operated small fractures, in contrast to the operative-only Frankfurt series [2]. Defect size is widely used as an objective parameter in surgical decision-making, and several groups have proposed CT-based frameworks: Burnstine [6] and Gart and Gosain [7] set out evidence-based criteria for isolated floor fractures, and Consorti et al. [8] proposed an operative algorithm based on standardized linear CT measurements that assigns approach and reconstruction material by defect type. The evidence underpinning specific numerical thresholds is, however, less settled than is sometimes assumed. Wevers et al. [9], in a systematic review of CT parameters in pure orbital wall fractures, found the relationship between individual measurements and both treatment choice and outcome to be inconsistently supported, with heterogeneous measurement techniques across studies.

We therefore report the distribution of defect sizes without proposing or endorsing a threshold. The 18.0% of patients with a sagittal diameter above 20 mm represents the upper end of the size distribution in this population; whether these patients were more likely to undergo repair was not analyzed, and no inference about operative indication can be drawn from the present data. The morphometric contribution of this study is the distribution itself, measured consistently across an unselected presenting cohort, which may serve as a reference denominator for future work that does examine thresholds.

### 4.4. Radiological Muscle Involvement and Diplopia

Content herniation was present on CT in 55.1% of patients, indicating that some degree of orbital content displacement is common rather than exceptional in this population. IRM involvement on CT was recorded in 14.7%. The interpretation of this figure requires care, and the distinction between radiological and clinical entrapment is the central caveat of this study’s clinical findings. Radiological muscle displacement does not establish mechanical incarceration; conversely, true entrapment may be present without being described on CT. Silverman et al. [10] reviewed 45 operatively confirmed entrapped floor fractures and found that radiology reports commented on possible entrapment in only 21 cases (47%), with no mention in the remaining 53%. Among the cases where entrapment was raised radiologically, 16 of 21 (76%) had the inferior rectus incarcerated at operation and 5 (24%) had orbital fat, indicating that a radiological impression of entrapment does not reliably distinguish muscle from fat.

In the present study, forced-duction testing was not performed systematically and dynamic motility assessment was not documented consistently, so no correlation between the CT finding and clinical restriction could be examined. The 14.7% figure should therefore be understood as the prevalence of a radiological descriptor across an unselected presenting population, not as the prevalence of surgically confirmed entrapment, and it is not directly comparable with rates from operative series. Establishing the correspondence between CT findings and intraoperative or forced-duction findings would require prospective data collection and is a priority for subsequent work from this cohort.

Diplopia at initial evaluation was documented in 17.6%, derived from the full presenting cohort rather than a symptomatic subgroup, and falls slightly below the 20–30% range reported in series drawn from operative or referred populations [10,12]. Acute diplopia has more than one cause: in smaller defects it may reflect muscle edema and hematoma and resolve spontaneously, whereas persistent diplopia is generally regarded as an operative consideration [6,7]. The present study recorded diplopia only at presentation and did not follow its course, so it cannot contribute to that distinction.

### 4.5. Concomitant Injury and the Pediatric Subgroup

Concomitant facial fractures in 27.9% of patients, principally zygomatic complex and nasal fractures, confirm that orbital injuries frequently occur as part of a wider facial injury pattern and support comprehensive assessment of the facial skeleton when an orbital fracture is identified [1,14]. Patients under 18 years comprised 13.6% (*n* = 203) of the cohort. The pediatric literature describes a distinct injury pattern in which the elastic immature orbital skeleton predisposes to trapdoor-type fractures [11,16,17]. The present analysis did not examine the pediatric subgroup separately: mechanism, fracture pattern, morphometry, radiological muscle involvement, diplopia, and management were not stratified by age group, and no pediatric-specific inference is drawn here. Nor were white-eyed blowout presentations or oculocardiac reflex events recorded as structured variables, so their frequency in this cohort cannot be reported. A dedicated analysis of the pediatric subgroup, using age-stratified modelling and clustering by patient, is in preparation and is reported separately; the pediatric figures are presented here only as part of the demographic description of the whole cohort.

### 4.6. Limitations

This study is descriptive and retrospective, and its principal limitations follow from that design. It does not evaluate management strategies or outcomes. Longitudinal variables, including postoperative motility, enophthalmos, and revision surgery, were recorded inconsistently and could not be analyzed; no conclusion about the effectiveness or optimal timing of any intervention can be drawn from these data.

The reliability of the CT morphometry was not formally assessed. Measurements were made by two experienced observers, but intraobserver and interobserver agreement was not quantified, and scanner hardware and acquisition parameters, including slice thickness, varied over the 22-year study period. Defect dimensions should therefore be read as consistently obtained by a small number of observers using a standardized technique, but without a demonstrated reliability coefficient. The elliptical area is a size index rather than a true surface area, as set out in Section 2.4.

Ascertainment of IRM involvement was radiological only, with no forced-duction or systematic motility correlation, as discussed in Section 4.4. Diplopia was recorded at presentation without follow-up. Selection is a further constraint: the cohort is drawn from patients referred to or managed by a single regional maxillofacial service, so patients with minor fractures managed elsewhere are underrepresented, and the 72.9% operative proportion reflects that referral pattern rather than the operative rate in the underlying population. The study is single-center, and although the size and diversity of the catchment population support relevance beyond the institution, generalization to centers with different trauma profiles, particularly with respect to the ballistic injury burden, should be made with caution. Finally, the absence of formal testing beyond the single pre-specified comparison means that all relationships suggested in this discussion remain untested hypotheses generated by, rather than demonstrated in, this cohort.

## 5. Conclusions

This study provides a descriptive epidemiological characterization of 1489 consecutive orbital fracture records managed at a level 1 maxillofacial trauma center over 22 years. The injury affected predominantly young adult males, with falls and interpersonal violence the leading mechanisms, and female patients were on average 12.4 years older than male patients at the time of injury. The orbital floor was the dominant fracture site, and CT-measured defects were distributed across the full size range, with the majority in the medium category. Orbital content herniation, radiological inferior rectus involvement, diplopia at presentation, and concomitant facial fractures were each common, indicating the diagnostic complexity of this injury pattern.

These findings describe the population presenting to a regional referral center. They do not evaluate treatment decisions, thresholds, or outcomes, and they should not be used to define surgical indications or optimal timing of repair. Their value is as a denominator: a consistently measured, full-spectrum institutional cohort against which subsequent outcome-focused and comparative analyses can be interpreted, including the operative versus non-operative comparison that this dataset will support once management indications and follow-up variables are completely recorded.

## Figures and Tables

**Table 1:** Demographic characteristics of the study cohort (*n* = 1489; September 2001–November 2023).

Characteristic	*n*	% or Mean ± SD
Total records	1489	100.0
Age (years), all	1489	36.5 ± 20.0
Median (IQR)	-	31 (21–51)
Range	-	1–98
Sex	1489	
Male	1171	78.6
Female	318	21.4
Male:female ratio	-	3.7:1
Age by sex		
Male	1171	33.9 ± 17.7
Female	318	46.3 ± 24.6
Age group		
<10 years	62	4.2
10–17 years	141	9.5
18–29 years	499	33.5
30–39 years	232	15.6
40–49 years	175	11.8
50–59 years	151	10.1
60–69 years	100	6.7
≥70 years	129	8.7
Broad categories		
Pediatric (<18 years)	203	13.6
Working age (18–64 years)	1118	75.1
Older adult (≥65 years)	168	11.3

IQR, interquartile range; SD, standard deviation. Age between sexes was the single pre-specified comparison (Mann-Whitney U test, *p* < 0.001).

**Table 2:** Fracture characteristics, defect morphometry, clinical findings at presentation, and timing of repair.

Variable	*n* Evaluated	% or Mean ± SD
Laterality	1489	
Left	813	54.6
Right	651	43.7
Bilateral	25	1.7
Fracture location	1489	
Orbital floor, isolated	873	58.6
Floor + inferior orbital rim	308	20.7
Floor + medial wall	87	5.8
Orbital roof	40	2.7
Medial wall, isolated	41	2.8
Multi-wall/other	140	9.4
Defect morphometry	1487	
Sagittal diameter (mm)	1487	13.7 ± 6.1
Range	-	1.2–28.5
Small (<10 mm)	441	29.7
Medium (10–20 mm)	779	52.4
Large (>20 mm)	267	18.0
Coronal diameter (mm)	1487	13.3 ± 5.1
Range	-	1.0–23.8
Estimated defect area (mm^2^, elliptical index)	1487	149.9 ± 94.0
Clinical findings at presentation		
Content herniation on CT	1489	55.1 (*n* = 820)
IRM involvement on CT	1489	14.7 (*n* = 219)
Diplopia at presentation	1489	17.6 (*n* = 262)
Concomitant facial fractures	1489	27.9 (*n* = 416)
Management and timing		
Operative management	1489	72.9 (*n* = 1086)
Non-operative management	1489	27.1 (*n* = 403)
Diagnosis to repair, days	1086	2.4 ± 2.3
Median (range)	-	2 (0–12)
Same day (Day 0)	141	13.0
1–7 days	906	83.4
8–14 days	39	3.6

Percentages for timing categories are of the operated subgroup (*n* = 1086). Estimated defect area is a standardized size index derived from two linear diameters, not a measured surface area (Section 2.4). IRM involvement on CT is a radiological descriptor and is not synonymous with surgically confirmed entrapment (Section 2.3). CT, computed tomography; IRM, inferior rectus muscle; SD, standard deviation.

## Data Availability

The anonymized data supporting the findings are available from the corresponding author upon reasonable request, subject to applicable institutional data-sharing rules and ethical approvals.
